# An examination of veterinarians’ negotiation of emotional labor

**DOI:** 10.1016/j.qrmh.2025.100001

**Published:** 2025-03-18

**Authors:** Lynsey K. Romo, April A. Kedrowicz, Katelin A. Mueller, Colin Mayhorn

**Affiliations:** a North Carolina State University, Department of Communication, 201 Winston Hall, Campus Box 8104, Raleigh, NC 27695-8104, United States; b Department of Clinical Sciences, North Carolina State University, 1060 William Moore Drive, Raleigh, NC 27606, United States

**Keywords:** Emotional labor, Emotion management, Veterinarians, Mental health, Wellbeing

## Abstract

Veterinarians are expected to care for animals while managing clients' emotions and dealing with stress, depression, burnout, anxiety, suicidal ideation, and other mental health struggles that accompany their profession. Through an open-ended qualitative survey of 124 alumni of a southeastern U.S. vet school, this study was designed to provide a rich, holistic examination regarding veterinarians' management of emotional labor. The investigation found that although veterinarians felt overwhelmed, frustrated, powerless, and fearful, they were institutionally expected to suppress these feelings, which they did by focusing on logic and facts over emotions and by reframing negatives into positives. The study also showcases how participants, by staying neutral and strong for themselves and clients, engaged in double-faced emotion management, newly applying this concept to veterinary medicine. Participants coped with emotional labor demands by turning backstage, where they sought support, allowed themselves to get emotional, engaged in self-care, and decided to enact tangible changes. Some participants were unable to truly find peace backstage, however, due to an entrenched veterinarian identity, lack of communication skills, or preexisting mental health struggles that made it difficult to disengage from their emotions. This study largely supports the claim that emotional labor may be worsening some vets’ preexisting stressors and mental health struggles and recommends that veterinary programs universally incorporate training that targets development of interpersonal communication competence, emotional labor, and wellbeing and their ability to decompress backstage.

## Introduction

Veterinarians are at four times higher risk of suicide than the general population (e.g., Tomasi et al., 2019). This is likely due to several factors. First, veterinarians must regularly euthanize animals (e.g., Hannah & Robertson, 2021). This death work (e.g., Morris, 2012) causes emotional and moral distress for both the veterinarian and the pet owner (Connolly et al., 2022, Dow et al., 2019, Scotney et al., 2015) and allows vets easy access to lethal drugs (Bartram and Baldwin, 2010, Witte et al., 2019). Additionally, veterinarians face high levels of stress, depression, burnout, compassion fatigue, work-life imbalance, anxiety (e.g., Pohl et al., 2022), and feelings of being undervalued (e.g., Coe et al., 2007).

Furthermore, veterinarians must break bad news (e.g., Nickels & Feeley, 2018; Tranberg & Brodin, 2023), have difficult financial conversations about medical costs (Coe et al., 2007), and encounter clients who cyberbully them (e.g., Teller, 2023), are unable or unwilling to pay for treatment (e.g., Coe et al., 2007), or are negligent or abusive towards their animals (Sanders, 1994). Veterinarians also work long hours (Pohl et al., 2022) for relatively low pay and high student loan debt (Sanford & DeBowes, 2024) and have to rush appointments due to time constraints (Belshaw et al., 2018).

Additionally, veterinarians are expected to engage in emotional labor–"management of feeling to create a publicly observable facial and bodily display…sold for a wage" (Hochschild, 1983, p. 7). Emotional labor is a known stressor (e.g., Connolly et al., 2022) and “taxing” (Hannah & Robertson, 2021, p. 2021) for veterinarians, who describe feeling “emotionally drained” and “worn out all the time” (Connolly et al., 2022, p. 374). Emotional labor has also been linked to burnout and dissatisfaction more generally (e.g., Wharton, 1999).

While it is known that vets engage in impression management and emotional labor (Connolly et al., 2022, Owens, 2015), studies on veterinarians’ experiences with emotional labor have been limited to specifically examining “death work” such as euthanasia, wherein vets try to regulate their emotions around highly emotional clients (Hannah and Robertson, 2021, Morris, 2012) or breaking bad news, when veterinarians find themselves “on stage” (Tranberg & Brodin, 2023, p. 1356), trying to build rapport and trust while being direct (Nickels & Feeley, 2018). While we know that veterinarians sometimes feel as though they are performing in front of patients (e.g., Owens, 2015; Tranberg & Brodin, 2023), lacking is a holistic sense of the emotions vets must express and/or suppress and how they negotiate emotional labor demands. Veterinarians’ high suicide rate and the “highly emotional” nature of veterinary medicine (Hannah & Robertson, 2021, p. 132) make the communication and mental health behaviors of this population even more important to examine, as emotional labor may be exacerbating vets’ stressors and mental health struggles (Connolly et al., 2022). After all, while emotional labor has been connected with job satisfaction in some industries (e.g., Zapf, 2002), it has been associated with negative physical and psychological health effects among healthcare workers (cf., Bagdasarov & Connelly, 2013). Thus, the goal of this study is to explore how veterinarians experience and communicatively cope with emotional labor. Below, we detail emotional labor and our methods before presenting the results of this qualitative study.

### Emotional labor

Emotional labor or emotion work (Hochschild, 1979, Hochschild, 1983) is a strategic form of impression management that builds on the dramaturgical perspective (Goffman, 1959), which maintains that people in the presence of others (the front stage) manage their impressions to create a desired public identity. Backstage (away from others), individuals can showcase their true selves. Emotional labor is fundamentally communicative and involves people expressing or suppressing feelings, outward emotions, and interaction in front of others as part of their employment (Hochschild, 1979, Hochschild, 1983). In other words, employees must manage their emotions through emotional display or expression rules (e.g., they must act happy or stay calm, masking true emotions through surface acting) as a condition of their jobs. Other employees are able to truly feel the desired emotions through deep acting (Hochschild, 1983). Beginning with research on flight attendants (Hochschild, 1983), emotional labor has been studied in such realms as teachers (Dunn, 2023), college athletes (Romo, 2017), and more recently, health care.

After all, medical professionals such as nurses, care workers, medical doctors, and hospital staff must incorporate emotional experiences into their care as a means of clinical decision-making and practice (e.g., Funk et al., 2017; Vinson & Underman, 2020). Healthcare professionals perform emotional labor regularly, through suppressing feelings, which in turn makes others feel cared for and safe (Erickson & Grove, 2008), and regularly empathizing with patients (Vinson & Underman, 2020). Emotional labor in healthcare contexts is related to numerous negative physical and psychological health consequences, such as decreased mental health and stress, burnout, anger, fear, sadness, and emotional exhaustion (cf., Bagdasarov & Connelly, 2013). However, on top of the work involved in managing their own emotions, medical professionals must regulate their own emotions while tending to their clients’ feelings. Managing two parties’ emotions simultaneously is known as “double-faced emotion management” (Tracy & Tracy, 1998). While not specifically studied in a veterinary context, double-faced emotion management typically occurs in high-stress settings (Tracy & Tracy, 1998). For example, 911 call center workers manage the emotions of callers (anger, hysteria, sadness) while also negotiating their internal feelings (e.g., irritation, disgust, amusement [Tracy & Tracy, 1998]). Veterinarians, too, are institutionally expected to manage their own and others’ emotions. For instance, they must suppress their anger, sadness, and disappointment while conveying compassion and empathy, assuaging client guilt, building and maintaining trust, and managing angry or grieving clients (Hannah and Robertson, 2021, Morris, 2012, Owens, 2015).

While we know that veterinarians enact emotional labor, research is limited on the sources of emotional labor and strategies enacted by veterinarians to manage the dual emotional labor demands of both their own and clients’ feelings. Because emotional labor can have mental health consequences, we posed the following research question: How do veterinarians negotiate the performance of emotional labor?

## Materials and method

Following approval from the Institutional Review Board (IRB), as part of a larger study, alumni of a highly rated veterinary school of medicine in the southeastern U.S. were recruited via email by the vet school to complete a veterinary wellbeing questionnaire through Qualtrics. Questionnaire data (which involved closed and open-ended items) were collected in August and September, 2023. As an incentive, at the end of the questionnaire, participants could choose to enter a raffle to win one of two $100 Amazon gift cards funded by the authors’ university.

### Participants and procedure

Consistent with other research investigating potentially sensitive information (e.g., Romo & Dailey, 2014), we deployed an online questionnaire. Questions involved several mental health topics, including stress, coping, finances, satisfaction, and work-life balance. Upon receipt of the survey link, participants electronically granted their consent before beginning the questionnaire. The consent form informed participants that if they were experiencing psychological distress at any point, they could stop the interview or skip any uncomfortable questions. Participants were also instructed they could text “HELLO” to 741741 to reach the National Crisis Hotline. Participants could also take a break from the questionnaire and return at their convenience. To protect participants' privacy, they were never asked for their name or to provide any identifying information. The researchers assigned pseudonyms to the responses after participants completed the survey.

While 139 vet school alumni responded to the survey; 15 did not answer the 20-question open-ended, qualitative portion of the questionnaire. Thus, our sample size consists of 124 participants who completed the open-ended portion. Consistent with the predominantly female veterinarian population (veterinary medicine has been dominated by women since 2009; Hopkins, 2024), 94 (75.8 %) of the participants were female. Participants’ ages ranged from 28 to 76 (*M* = 49.4; *Mo* = 62). One hundred fourteen (91.9 %) of the participants identified as Caucasian/White, two (1.6 %) as Asian, one (.08 %) as Black, one (.08 %) as American Indian, and six (4.8 %) as having a mixed race. Ninety participants (72.6 %) were married/partnered, 26 (20.9 %) were single, five (4 %) were divorced, two (1.6 %) were widowed, and one participant chose not to respond. The participants' number of children ranged from no children (*n* = 47; 38 %) to four children. Seventy-five (60 %) had children, and two participants chose not to respond to that question. A majority of participants worked full-time (*n* = 100; 81 %) with only 19 participants working part-time (15 %). Participants’ area of veterinary medicine included private practice (*n* = 72; 58 %), corporate practice (*n* = 20; 16 %), other (*n* = 17; 13.7 %), academia (*n* = 11; 9 %), retired (*n* = 3; 2 %), and private lab (*n* = 1;.08 %). The participants' practicing region included a suburban region (*n* = 72; 58 %), an urban region (*n* = 22; 18 %), a rural region (*n* = 21; 17 %), or none of the above (*n* = 9; 7.3 %). The vast majority of participants (*n* = 109; 88 %) were practicing veterinarians at the time of data collection.

### Data analysis

The first two authors analyzed the questionnaire’s qualitative responses via reflexive thematic analysis (Braun and Clarke, 2006, Braun and Clarke, 2019). We first gained familiarity with the data by reading through each participant’s responses. We first engaged in open coding by reading through each response and highlighting keywords and phrases. As feelings of frustration and powerlessness repeatedly appeared throughout the data, we then returned to the data using emotional labor (Hochschild, 1979, Hochschild, 1983) as a lens to understand how veterinarians negotiated the performance of emotional labor. In this round of coding, we created coding categories and subcategories that specifically focused on isolating the emotions experienced by the veterinarians and how they were (un)able to manage their and their client’s emotions. We then collapsed this data into themes, which we reviewed before defining and naming them and identifying exemplars (the participants' own words) from the data. To preserve the integrity of participants’ responses, we did not edit the examples for grammar or punctuation errors.

## Results

### Theme 1: Emotional experiences of veterinarians

Consistent with the struggles and stressors facing veterinarians (Connolly et al., 2022), this study found that veterinarians experienced several strong emotions. Unlike existing research, however, which found that veterinarians felt anger, sadness, and disappointment (Morris, 2012), the emotions that cut across our participants' voices were predominantly feeling overwhelmed, frustrated, powerless, and fearful.

#### Feeling overwhelmed

The vast majority of veterinarians spoke of feeling overwhelmed by client needs, identity challenges, and lack of time.

First, many participants expressed feeling overwhelmed by clients' "unrealistic demands," as Mia put it, and Lea explained that she was overwhelmed by “managing people and their expectations, as well as solving their problems.” Similarly, Rachel said that clients' ignorance and insistence contributed to these feelings:

I do not think people understand the stresses of our job. And I do not think they understand they are not the only person with a sick pet. Everyone these days demands same day appointments, wants the doctor to call them back right away, wants immediate gratification and immediate cures.

Similarly, Scott maintained: "The entire industry seems to be overwhelmed with too many requests and entitled clients who seem to want everything done right away. People can be so hurtful.” Additionally, Marsha explained clients' "expectation that we should always be available to them no matter what the clinic hours are" was incredibly overwhelming.

Participants also talked about how identity struggles contributed to these feelings. As Ivan explained, “The job has always come first, and my family has understood and accepted that. My identity is tied up in my profession.” Several participants echoed Sophia who talked about how “It’s really challenging to have so much of my identity tied into it—not just personally but the way the public and my friends view me.” Ava said even her “friends see me as a vet first.” Many veterinarians felt like they “can never be totally off” (Sophia) and that being a veterinarian was more than a job: “it's a lifestyle or basically a life” (Nancy). Because those who strongly identified as a vet both at work and at home were continually in veterinarian mode, they struggled even more with feeling overwhelmed.

A lack of time also contributed. As Amber explained, veterinarians like her “can't do the amount of work I need to do in the amount of time available.” Furthermore, vets were “pulled in too many directions—not enough time in the day for client communication” (Patricia). They did not have time to “adequately care for patients in time allotted” (Skylar). Far too often, appointments were rushed. Additionally, participants such as Caitlin said despite time constraints, clients still expected to talk with their veterinarian “on the phone for 30 min any time.” Participants also stated that some of these time restrictions arose from staffing shortages, For instance, Sue discussed “the constant churn of being understaffed,” and Phoebe stated that the “inability to maintain trained and competent staff adds unnecessary stress and decreases job satisfaction.” As Harriet remarked: "The need for ER practitioners is so great that if I say no to relief shift, there is a very good chance that the hospital will close and pets will die." Additionally, Dan shared, “Me and the few other practice owners in our county can't get people to come work for us so we are the only ones to care for the thousands of pets in the communities.” The lack of time made participants feel overwhelmed and contributed to their stress.

#### Feeling frustrated

Participants also reported feeling frustrated with how clients treated veterinarians and their animals, as well as the lack of communication training veterinarians received in veterinary school.

Many participants felt frustrated with clients. Some described them as “asshole entitled clients” (Justine) and “difficult (pushy, mansplaining, clueless) clients” (Patricia). Skylar bluntly said she was frustrated by the “ignorance, apathy, insolence, arrogance and cruelty of people, repeated daily in my face." Participants were especially frustrated with clients who dismissed their expertise ("turning to Dr. Google, their breeder, neighbor, etc.” instead of vets, in the words of Wendy) or were reluctant to pay for treatment. Wendy went on to recount the irony of people who “pay a lot of money for a mutt (poodle x) and then can't pay $30 for a vaccine that will help prevent a potentially deadly disease.” Similarly, Phoebe shared her frustration over:

Owners wanting that silver bullet but not wanting to pursue the necessary work up or give the condition the necessary effort, with medical and especially BEHAVIOR cases. I do not have a crystal ball or wand. I will do my best with the information provided. This is a TEAM sport.

Relatedly, others, like Morgan, were frustrated by “how much time is spent convincing clients of the necessity of treatment versus actually treating patients.” Marsha, who described clients as “angry and demanding,” had similar feelings to Stevie, who added that clients are “people unwilling to spend money on their pets, unwilling to follow up and want ‘quick fixes’” or who seek “miraculous outcomes (often with a minimal investment),” as Ethan said. As Nancy lamented: "People want so much and don't accept that it may not be possible or an answer may not be obtained."

Relatedly, participants overwhelmingly felt frustrated by what they perceived as the general public’s disrespect and devaluing of their profession. As Pam observed, “Clients don't respect you like they respect human physicians.” Ethan found it frustrating when a "client is unable or unwilling to understand what our limitations are or will not recognize the value of what I am providing." When asked if she would become a veterinarian again, Maria reflected this disrespect, saying: “No, it's mentally draining, unrewarding, not cost-effective and overly stressful, not respected.” Other participants, such as Izzie expressed how “it’s exhausting always being the asshole or taken for granted.”

Participants also felt frustrated with the lack of communication training they received in veterinary school, as many reported they were unprepared for communicating with clients, an important part of doing their jobs and providing animals with proper care. As Lea said: “Interpersonal communication is hugely important, and it was not emphasized during my curriculum.” Furthermore, Delaney stressed that there needed to be “more time on client communication." Furthermore, Marsha described wishing there had been “more communication type courses and navigation [of] difficult conversations,” and others talked about a greater need for instruction on navigating conflict. As Harriet said: “We should have gotten A LOT more professional development curriculum about communicating with each other and clients, wellness, career planning, perfectionism, imposter syndrome, etc.” Participants expressed a sense that they were left to navigate real-world communication largely on their own, which was incredibly frustrating and stressful for them and made practicing as a veterinarian much more difficult.

#### Feeling powerless

Participants regularly voiced feeling powerless, particularly involving influencing client decisions about treatment options, treating animals, and saving animals’ lives.

Many participants talked about how powerless they felt when trying to persuade a client to pay for treatment for their animal. Indeed, client finances resulted in many participants grappling with the “cost-of-care outpacing owner's ability to pay,” as Frank observed. Debra also reflected on this lack of control, sharing:

Very often the people have few options. I sometimes can get a charity to pick up the bill, but not always. When an animal's life boils down to how much money their humans can afford, this can be very depressing.

Irene further exhibited this sense of powerlessness, stating that veterinarians have “the ability to help, but hands are tied by finances or other client factors.” Similarly, “I hate being bound by owner restraints,” David said.

Additionally, other participants felt powerless due to their inability to treat animals due to schedule and clinic constraints. As Rachel expressed: “the feeling of not wanting to turn an animal away but no room in schedule.” Hank, 56, further said, “You can't help everyone all the time.” Participants felt this emotion even more strongly regarding saving animals’ lives. A reality for all participants was that even with the best medicine, their patients were ultimately going to die on their watch. For instance, Ivan reflected on “the short life span of my patients - I have a 100% mortality rate for the ones I started with. For many people losing their pet is like losing a member of the family.” Similarly, Amaya voiced, “You can do everything right and your patient can still die." Conversely, Ethan stated, “I had to become comfortable (still working on this) with the reality that I can do everything right and still sometimes end up with negative outcomes, just due to the variability of so many factors.” Participants acknowledged the difficulty of an animal's life often being out of their control. Some participants struggled with not taking this powerlessness as a personal failure. As Michael said, many veterinarians "are more likely to be easily stressed by failure and therefore prone to depression and unable to face failure and death.” Powerlessness hit some participants particularly hard.

#### Feeling fearful

Participants also felt fearful about performing poorly, as well as potential aggression from clients.

Participants, particularly those who had been practicing fewer years, commonly shared their fear regarding determining the correct diagnosis and treatment plan for their patients. As Tabitha explained, “At times I can feel anxious about whether I am giving them the best options or information to make their decision,” and Samantha acknowledged fear of “not knowing if what I am doing is the absolute best treatment.” Similarly, Ella said, “Knowing there are specialists that would probably handle a case differently than how I would instinctively handle it made me feel like I wasn't the best doctor to be treating that patient.” Rachel said she was worried that, “I've failed if someone doesn't like me or my style of practice.” Relatedly, participants such as Karen acknowledge that “imposter syndrome was a huge issue for me.”

Other participants were fearful about how they would be treated by clients on social media. As Daphne explained, she had a “fear of being bullied on social media because an owner didn't get the outcome they desired for whatever reason.” Indeed, the “fear of unfair reviews/comments online” as Opal put it, was prominent among participants, consistent with research on veterinarian cyberbullying (e.g., Teller, 2023). As Wendy stated, “Society is different these days. It makes it a lot harder to practice good medicine with threats of lawsuits and social media storms." Furthermore, Emily said the “negative comments or posts from clients are very detrimental to wellbeing.” A few participants, such as Pam, even feared more extreme forms of client aggression: “I worry they will shout/scream/blame me or even become physically violent (have seen it happen before).”

### Theme 2: Institutionally mandated emotional expressions

Consistent with existing veterinary research (Hannah and Robertson, 2021, Morris, 2012, Owens, 2015), participants frequently were required to suppress their feelings and emotions, particularly during interactions with clients. The current study found that veterinarians' mandated expressions, which were contextually performed as an unspoken part of the job, involved staying neutral and being strong for self and clients and engaging in double-faced emotion management, especially when having difficult conversations surrounding finances, end-of-life, or breaking bad news.

#### Staying neutral and strong for self and clients

As Harold acknowledged, veterinarians “have to absorb a lot of negativity and sadness” that often, in Brandy’s words, “weigh heavy” on them. Many participants described vet medicine as “emotionally demanding” (Marsha). Similar to college athletes, who were expected to emit mental toughness (Romo, 2017), veterinarians had to “stay detached from the situation” (Bailey) and, as Amaya said, “divorce myself from my personal emotions without being seen as distant or uncaring.” Betty was able to detach by “focus[ing] on being objective instead of the impact it will have on the family.” Furthermore, like Laurence, who said he was able to "disconnect emotionally," Bailey voiced, “I just try to stay calm and carry on at work. Do the best I can.” Similarly, Wendy said, “I feel like my emotions turned off a long time ago, and I compartmentalize everything, good and bad.” As Robert stated, “The short-term conversations with patients such as end of life and breaking bad news do not affect me a lot because I can't allow it to.” Many participants learned to “build a wall mentally” (Debra).

Another way participants were able to suppress their feelings of sadness, powerlessness, and frustration was by reframing these negative emotions. For instance**,** Opal shared that when interacting with clients, “I just keep telling myself that this person is trying their best and focus on positive intent and that makes it much easier.” Similarly, Sue acknowledged that while, “it affects my wellbeing when I deliver bad news, it is a privilege to assist people with difficult life decisions.” Correspondingly, Ethan stated, “I love being a vet! Every day is different! Yes, there are challenges but do you want a life without challenges?” Reframing by focusing on the benefits versus the costs helped participants display institutionally mandated emotions.

#### Engaging in double-faced emotion management

While veterinarians had to keep their own emotions in check, they were also expected, as Bailey added, to “offer compassion, understanding and help for my clients.” In this way, veterinarians engaged in double-faced emotion management. Consistent with other research (e.g., Tracy & Tracy, 1998), participants acknowledged they had to simultaneously manage their emotions and their clients’ emotions. Indeed, a considerable component of the participants’ emotional labor involved comforting and consoling clients, with some participants surprised at “how much of a therapist I am to clients” (as Sierra put it), stating, in Karen's words, that they “never get an emotional break at the end of the day.”

The vast majority of participants spoke of having to display empathy as part of their job: in Morgan’s words: “Empathy to connect with clients, validating their emotions, helping them explore options.” As Mary said, “I usually approach [challenging conversations] with a lot of kindness and reflective listening.” Additionally, as Ella put it:

I try to be very compassionate and treat/talk to owners as I would want to have done to me or my family. Having this approach helps me mentally because I know not all veterinarian[s] show compassion, and that is one gift I can give to them in challenging times.

Other participants performed emotional labor by centering conversations around examples and information, making communication more fact- as opposed to emotion-based. This technique helped them manage both their own and clients' emotions. For instance, John stated:

I use personal experiences with my pets or previous experiences with clients/pets at work to give personal examples. These conversations are honest and true and therefore don’t affect my wellbeing negatively. I can’t and won’t save every pet for a variety of reasons. My goal is to be honest and fair in every interaction. If I do that, I have succeeded.

Additionally, Natasha said: “I find that making matter of fact statements that do not convey judgment or expectation makes it easier for both parties to navigate.” Similarly, Samantha expressed:

My goal is to be upfront yet gentle and give the owners multiple options including potential next steps or outcomes based on these different options. I feel better if, even if the owner doesn't opt for the best treatment or what I personally would have done, that they seem informed and ok with their decision.

Relatedly, Sarah stressed the value of information over emotions, saying, “Remember that clients are scared and worried and have no baseline knowledge. Most difficult conversations can be diffused with a good explanation.” Additionally, Imogine said that her approach was to “Go slow and make sure the client knows that there are options (even if they are not ideal).” As Cherry maintained, “I try to discuss events, diagnosis, options, etc. ahead of time and plan ahead so clients are not as blindsided or upset and therefore neither am I.” Providing clients with reasonable explanations and information helped veterinarians regulate their own emotions while also helping clients manage theirs.

### Theme 3: Communicatively coping with emotional labor

Consistent with the literature (e.g., Funk et al., 2017), participants widely expressed that they turned backstage, away from public view, to escape the demands of the profession and emotional labor. In private, participants sought support, allowed themselves to get emotional, engaged in self-care, and made the decision to enact tangible changes.

#### Seeking support

One of the major ways participants coped with emotional labor was by communicating backstage. This strategy largely involved seeking support from trusted others with whom they could let down their guard. Such “bitch sessions” (Izzie) or “venting” (Sandra) with friends, family, and colleagues, provided participants with some peace. As Rose explained, “My friends are a great support network, they listen to me.” Similarly, Natasha stated, “I vent to my husband about it in private, and then I move on.” Other participants turned to counseling and religion for support. For instance, Evie described “seeking professional counseling for burnout" and many participants turned to prayer. Austin said he “prays every day multiple times when I'm overwhelmed.” Jessica similarly expressed, “There are hard days, but I do not have to fix everything or solve all problems—I can daily choose to lay my burdens down with my Lord.”

#### Allowing themselves to get emotional

Two participants admitted to letting themselves break down in private. Crying occurred at home and also at work, away from clients. For example, as Rose said, "For euthanasias, I take a minute or two to cry if needed before going into the next appointment." Similarly, Debra said that after being accused by clients of "being in it for the money," "I go home and cry." This strategy is consistent with nurses who waited until they were home to cry and grieve (Funk et al., 2017), self-soothing backstage.

#### Engaging in self-care

Participants also engaged in self-care techniques backstage. These coping strategies included maintaining a healthy diet, exercise, and sleep routine, taking anxiety and depression medication, and engaging in hobbies. Many participants expressed the positive effect exercise had on their wellbeing as a coping strategy. For example, Debra emphasized the importance of “regular, scheduled exercise” in improving her wellbeing, whereas Daphne stressed the need to work out “5–6 days weekly” as necessary for her mental health. Furthermore, Julie stressed the importance of “getting enough sleep and time to myself—not getting this makes it harder to deal with [stress].” An additional form of self-care included engaging in hobbies as a way to reconnect with their true selves and emotions backstage. As Luna said, “My wellbeing is enhanced by taking care of myself-spending time doing my hobbies and the things that make me happy, taking care of my body, and mind.” Participants’ hobbies included journaling, running, depth breathing, acupuncture, spending time with participants' own animals, and traveling. As Phoebe expressed: “Take that vacation-work will be there when you get back. Do things for yourself.” Similarly, Kaylie said, “When I’m over the top, I leave town & go to a fabulous hotel & get sucked up to big time!”

It is also important to note that some participants engaged in maladaptive coping, in which they coped with stressors in non-healthy ways. Examples included participants speaking of misusing alcohol or emotional eating backstage. While participants acknowledged that these ways of coping “are really bad for you” (Harold), they found that drinking, overeating, or emotional eating helped them deal with emotional labor demands by numbing their stressors. These techniques, while technically negative, helped participants numb their feelings, providing them an escape from emotional labor demands (Dalton, 2024, Metzger et al., 2017).

#### Enacting tangible changes

Some participants realized backstage that they needed to implement tangible changes to minimize and better cope with emotional labor pressures. These changes included switching employers, working fewer hours (Katie even left the profession and became a high school science teacher), and/or setting boundaries. For instance, as Eleanor explained, “I changed jobs twice in three years and fortunately found a job that works with my wellbeing rather than against it.” Other participants chose to work fewer hours as a means to lessen emotional labor burdens. As Luna said, “I am working less hours, so I have had a lot more time to prioritize myself and my life.” Relatedly, participants set boundaries to put a barrier between their professional and personal lives. Boundaries included “saying no to extra work” (Amber), “shutting off my work email and all work when I get home” (Lisa), "turn[ing] my phone off" (Justine), or “cut[ting] myself off from work to be with family” (Tabitha).

However, despite their best intentions, some participants noted how they grappled to disconnect from work, even backstage. As Morgan noted, “I try to give 100% at work so I don’t fret/obsess when I’m home.” However, she went on to acknowledge that, “Being an owner makes it difficult at times to truly get away from work; even on my days off I get calls.” Similarly, as Brandy voiced, “Being on call and my work hours and inability to truly ever leave work at work definitely impacts ability to manage stress.” Furthermore, as Ava said, “Having my identity be wrapped up in my career—not just in a professional setting, but having family and friends see me as a vet first. It can be isolating.”

A lack of a true backstage was even more prominent for participants who tended to ruminate or had preexisting mental health issues. For example, Samatha struggled to “leave work at work and not stress or think about it at home, either anticipating bad stuff that is going to happen the next day or agonizing over something that happened that afternoon.” Similarly, Natalie said, “I just want my time at home to be my time at home and not have to worry about cases.” And as Amaya stated, “You don't want to come into this field with a lot of emotional baggage…the weight of what I have carried with me for so long has taken its toll.”

The stress of always “being on,” the expectation to engage in double-faced emotion management despite feelings of powerlessness, frustration, and fear, and the other mental health stressors that accompanied their profession made it difficult for some participants to truly relax, even in private. Those with prior mental health challenges found a true backstage to be even more elusive, as being a vet intensified their struggles. As Amaya remarked, “Pre-existing depression worsened during vet school and ever since, to an alarming degree; I should have been more proactive in dealing with it.” Similarly, Sophia said, “I wish I had focused on overcoming imposter syndrome and anxiety before going to the practice.” These participants particularly struggled to cope with emotional labor demands.

## Discussion

Through an open-ended questionnaire of 124 alumni of a top-ranked veterinary school of medicine, this investigation provides a holistic sense of the emotions vets must express and/or suppress, and how they negotiate emotional labor demands involved in managing their own and clients' emotions. Existing literature has largely examined emotional labor in the context of euthanasia and breaking bad news, finding that veterinarians must suppress their anger, sadness, and disappointment (Hannah and Robertson, 2021, Morris, 2012, Owens, 2015). The current study examined veterinarians' emotional labor more holistically and richly, uncovering that participants frequently felt overwhelmed, frustrated, powerless, and fearful in regular interactions. These emotions–which participants were expected to suppress—provide valuable insight into the daily stressors veterinarians must contend with as everyday parts of the profession.

Reinforcing previous research, which found that veterinarians were required to convey compassion and empathy, assuage client guilt, build and maintain trust, manage angry or grieving clients, and be direct, the current study found that participants also had to stay neutral and show compassion and empathy for clients as institutionally mandated emotional expressions. Extending existing literature, this investigation provides insight into how participants tried to regulate their emotions–specifically being able to detach by focusing on logic and facts over emotions and by reframing negatives into positives to better help themselves suppress their emotions.

This study is also unique in that it showcases not just that veterinarians were experiencing emotional labor, but how they were able to cope with these demands. Consistent with other emotional labor research (e.g., Funk et al., 2017), many participants turned backstage for comfort and decompression. By seeking support, letting themselves get emotional, engaging in self-care, and deciding to enact tangible changes (such as switching employers, working fewer hours, setting boundaries), many participants were able to get some relief from the stressors of emotional labor. However, the study also uncovered that some participants, due to their strongly entrenched veterinarian identity, preexisting mental health struggles, or even lack of communication skills, may not have a true backstage available, where they can relax and separate themselves from the stressors of their profession. Similarly, some participants’ engagement in maladaptive coping behaviors—while useful for temporarily disengaging from emotional labor demands—is likely to lead to negative outcomes (e.g., Dalton, 2024; Metzger et al., 2017).

This study also extends the concept of double-faced emotion management (e.g., Tracy & Tracy, 1998) to a new context, providing language for the process by which veterinarians had to regulate their own and clients' emotions. This dual emotional labor reinforces research that, for people in the medical field, including veterinary medicine, emotional labor in and of itself served as an additional stressor that made it more difficult for participants to perform their jobs. Unlike some careers in which emotional labor can have beneficial consequences (Zapf, 2002), findings largely support the claim that emotional labor may be worsening some veterinarians’ stressors and mental health struggles (Connolly et al., 2022), particularly for veterinarians with a lack of preparation and training to guide their interactions with clients and manage the rigors of the profession.

## Practical applications

Several participants emphasized the lack of education they received about communication in general (particularly related to talking with clients and managing conflict and difficult conversations) as leaving them unprepared to navigate the emotional and mental health challenges of the profession. Although the participants' veterinary school currently offers a robust competency-based curriculum, including training in communication, collaboration, wellbeing, business, and professionalism, this training only began in 2014 with the first class graduating in 2018. As the average participant was 49.4 years old at the time of the study (with a mode of age 62), the majority of participants likely did not have access to the training as it had been implemented after they graduated. Thus, this study's findings suggest that these classes should continue to be required and mandatory at veterinary schools around the world. While most, if not all, veterinary programs must demonstrate graduates’ ability to communicate to remain accredited by the American Veterinary Medical Association (American Veterinary Medical Association, 2024), it is likely that the breadth and depth of this training varies across institutions, as no standardized curriculum is required.

This communication training should specifically prepare and educate budding veterinarians about the demands of emotional labor as part of providing a realistic job preview. Such a curriculum should provide information related to the realm within which veterinary medicine is practiced, including private versus corporate practice, time constraints, and clients’ contextual factors. Additionally, the training should help vet students build skills in relationship-centered care, collaboration in the veterinary medical team, competent communication, and building a professional identity. This training would help prepare future veterinarians to navigate these additional veterinary stressors (Pohl et al., 2022).

The curriculum should also train future veterinarians to decompress and build resiliency backstage by emphasizing healthy habits such as work-life balance, diet, and exercise, while focusing on the value of social support, therapy, and making tangible career changes when necessary. After all, as Jack said, the vet school’s “admissions process selects for type A people that are high stress and competitive to begin with.” It is possible that people enter vet school and the profession in even greater need of skills to help handle the emotional rigors of the profession due to the high standards they hold themselves to and the pressure they have already experienced. Similarly, as some participants mentioned that being a veterinarian worsened their preexisting mental health struggles, emotional labor training would help counteract these mental health risk factors while also serving as a transformative communicative force in vet medicine and veterinarian wellbeing.

In addition to receiving emotional labor training and mental health support in veterinary school, continuing education classes should emphasize managing emotional labor, seeking mentoring and ongoing mental health assistance, and peer support. Not only would this training be invaluable for practicing veterinarians, it would also ease the transition into the workplace for new graduates, since their colleagues would share a similar understanding of the importance of communication and social support in navigating emotional labor and enhancing wellbeing.

## Limitations and future directions

As the surveys only captured participants' general reflections, it is unclear how they experienced and managed emotional labor over time. Also, while an online questionnaire was effective in meeting the goals of the study in a less face-threatening manner than interviews or focus groups and also enabled them to complete the survey on their own time, like with other qualitative questionnaires (e.g., Romo & Dailey, 2014), the richness of the data may have been compromised in an effort to best accommodate participants. For example, as opposed to an in-depth, semi-structured individual interview, the authors were unable to follow up with participants or ask for clarification on responses.

Several future research directions exist. First, it would be valuable to parlay these qualitative findings into quantitative research to track veterinarians’ emotional labor over time to see how coping strategies (including maladaptive coping) and emotional labor demands evolve over their careers. Such research would allow for the quantification of the degree of emotional labor expended under specific circumstances and how emotional labor affects coping strategies, enabling the exploration of the relationships among variables. Additionally, since most participants completed their veterinary training before the implementation of the communication and professionalism curriculum, it would also be valuable to gather additional data to explore how participants' experiences with emotional labor may differ after having participated in a curriculum designed to prepare them for collaborating with team members and clients, attending to their own and other’s wellbeing, and navigating identity challenges. Furthermore, while veterinarians (including this study’s participants) largely work in small animal private practice (American Veterinary Medical Association, 2023), it would be useful for another investigation to specifically explore and compare the emotional labor comprising veterinary work across equine medicine and food animal/production animal medicine. Differences in context, clients, and constraints are likely to affect veterinarians’ emotional labor, experiences with stress, and impacts on professional identity.

## Conclusion

Framed by emotional labor, this study builds on existing research on the stressors facing veterinarians by richly showcasing how participants both engaged with and coped with emotional labor demands. The investigation extends double-faced emotion management to a new context while suggesting that emotional labor worsened some vets’ preexisting stressors and mental health struggles. This study recommends that veterinary programs universally incorporate training that targets development of interpersonal communication competence, emotional labor, and wellbeing to better support veterinarians’ enactment of emotional labor and their ability to decompress backstage.

## CRediT authorship contribution statement

**Lynsey K. Romo:** Conceptualization, Data curation, Formal analysis, Investigation, Methodology, Project administration, Supervision, Writing – original draft, Writing – review & editing. **April A. Kedrowicz:** Conceptualization, Writing – review & editing. **Katelin A. Mueller:** Data curation, Formal analysis, Methodology, Project administration, Writing – original draft, Writing – review & editing. **Colin Mayhorn:** Formal analysis, Writing – review & editing.

## Declaration of Interests

The authors declare no potential conflict of in terest, and all authors confirm accuracy.
